# Phosphoethanolamine-N-methyltransferase is a potential biomarker for the diagnosis of *P*. *knowlesi* and *P*. *falciparum* malaria

**DOI:** 10.1371/journal.pone.0193833

**Published:** 2018-03-05

**Authors:** Robert G. E. Krause, J. P. Dean Goldring

**Affiliations:** Biochemistry, University of KwaZulu-Natal, Pietermaritzburg, South Africa; Universidade Federal de Minas Gerais, BRAZIL

## Abstract

**Background:**

*Plasmodium knowlesi* is recognised as the main cause of human malaria in Southeast Asia. The disease is often misdiagnosed as *P*. *falciparum* or *P*. *malariae* infections by microscopy, and the disease is difficult to eliminate due to its presence in both humans and monkeys. *P*. *knowlesi* infections can rapidly cause severe disease and require prompt diagnosis and treatment. No protein biomarker exists for the rapid diagnostic test (RDT) detection of *P*. *knowlesi* infections. *Plasmodium knowlesi* infections can be diagnosed by PCR.

**Methods and principal findings:**

Phosphoethanolamine-N-methyltransferase (PMT) is involved in malaria lipid biosynthesis and is not found in the human host. The *P*. *falciparum*, *P*. *vivax* and *P*. *knowlesi* PMT proteins were recombinantly expressed in BL21(DE3) *Escherichia coli* host cells, affinity purified and used to raise antibodies in chickens. Antibodies against each recombinant PMT protein all detected all three recombinant proteins and the native 29 kDa *P*. *falciparum* PMT protein on western blots and in ELISA. Antibodies against a PMT epitope (*P*LENNQYTDEGVKC) common to all three PMT orthologues detected all three proteins. Antibodies against unique peptides from each orthologue of PMT, *Pf*CEVEHKYLHENKE, *Pv*VYSIKEYNSLKDC, *Pk*LYPTDEYNSLKDC detected only the parent protein in western blots and *P*. *falciparum* infected red blood cell lysates or blood lysates spiked with the respective proteins. Similar concentrations of *Pf*PMT and the control, *Pf*LDH, were detected in the same parasite lysate. The recombinant *Pf*PMT protein was detected by a human anti-malaria antibody pool.

**Conclusion:**

PMT, like the pan-specific LDH biomarker used in RDT tests, is both soluble, present at comparable concentrations in the parasite and constitutes a promising antimalarial drug target. PMT is absent from the human proteome. PMT has the potential as a biomarker for human malaria and in particular as the first *P*. *knowlesi* specific protein with diagnostic potential for the identification of a *P*. *knowlesi* infection.

## Introduction

The *Plasmodium* genus includes over a hundred species that infect vertebrate hosts including birds, rodents, reptiles, amphibians and simians, by dipteran vectors [1]. Four *Plasmodium* species, *P*. *falciparum*, *P*. *vivax*, *P*. *ovale* and *P*. *malariae* are infective and transmissible to humans by natural mosquito bites. Thanks to control efforts, human malaria has been restricted to tropical and subtropical regions where it remains endemic primarily due to Anopheline mosquito habitats. An estimated 148–304 million malaria cases occurred in 2015, resulting in 429 thousand deaths [2]. A fifth species, *P*. *knowlesi*, known to be experimentally infective to humans [3], was not regarded as naturally transmissible to humans despite a case noted in 1965 [4]. In 2004, however, Singh *et al*. [5] detected *P*. *knowlesi* infections in a human population in Malaysia and subsequently *P*. *knowlesi* has been included as the fifth human infecting species [6]. It remains unclear if natural transmission between humans is common and the species is still considered a zoonosis [7–9].

Since the Singh et al. study [5], *P*. *knowlesi* has been identified as the main cause of malaria in Malaysia [10], with positive diagnoses reported in Cambodia, Indonesia, Myanmar, Philippines, Singapore, Thailand, Brunei, Vietnam and the Nicobar and Andaman islands of India [11–18]. Only Laos and East Timor remain unaffected in the Southeast Asia region [19]. Since Fong et al. demonstrated experimental human-to-human transmission in 1971 [20] the asymptomatic human infections recorded by Fornace et al. [21] may present an additional reservoir to the natural monkey infections. This makes malaria elimination in Southeast Asia difficult as it would entail eliminating *P*. *knowlesi* from both hosts. The *A*. *leucosphyrus* group of mosquitoes that transmit *P*. *knowlesi* infections, actively feeds outdoors, making conventional vector control measures less effective [9, 22]. Fortunately *P*. *knowlesi* remains in Southeast Asia for the time being as there are no known *P*. *knowlesi* carrying *Anopheles* vectors beyond the region. *P*. *knowlesi* cases in travellers returning to Europe, USA and Australasia have been reported [22]. A changing global climate may also affect the vector distribution as new suitable habitats may arise [23]. Accurate diagnosis of *P*. *knowlesi* infections is a critical tool for treatment and to understand the dynamics of this species and its impact on human populations within Southeast Asia and in visitors returning home from this region.

Genomic evidence suggests the *P*. *knowlesi* infections in Southeast Asia were present in wild macaques prior to human settlement [7]. The morphological similarity between *P*. *knowlesi* and the late blood stages of *P*. *malariae* and the early trophozoite stages of *P*. *falciparum* leads to misdiagnosis and allowed *P*. *knowlesi* infections to escape detection [3, 24, 25]. Minor morphological differences between early trophozoite and late schizonts of *P*. *knowlesi* and *P*. *malariae*, can be identified in well stained thin blood film slides under careful examination by an expert microscopist [25, 26]. Busy routine diagnostic laboratories often only examine thick blood films and large numbers of slides, increasing the chance of misdiagnosis [27]. It has therefore been recommended that in *P*. *knowlesi* endemic regions, microscopic identification of *P*. *malariae* be diagnosed as *P*. *knowlesi*/*P*. *malariae* [26]. Importantly, prompt treatment of *P*. *knowlesi* infections is essential to prevent the onset of severe disease due to its short (24 hour) red blood cell cycle [28]. *P*. *knowlesi*, fortunately remains sensitive to chloroquine [29] and is highly sensitive to artemisinins [9] making treatment of the disease simple if diagnosed quickly and accurately. Misdiagnosis as a mild *P*. *malariae* infection (72 hour red blood cell cycle) may delay such treatment [5] resulting in the onset of severe disease and potential fatality [28]. *P*. *knowlesi* was shown to be three times as likely as *P*. *falciparum* to cause severe infections [30], emphasising the need for a rapid test capable of detecting *P*. *knowlesi* infections at point-of-care.

Diagnosis is essential for appropriate treatment, conserving resources and prevention of fatal malaria infections. Today the WHO recommends confirmative point-of-care malaria diagnosis prior to drug treatment for malaria, to improve treatment efficacy and limit the selective pressure for antimalarial drug resistance [31]. Malaria diagnosis has evolved to encompass microscopic to molecular biology based methods [32, 33]. One of these methods, immunochromatographic separation and detection of proteins with antibodies on rapid diagnostic test (RDT) devices allows for point-of-care diagnosis in a field setting. RDTs are cheap, rapid and easy to perform and interpret [32]. The first malaria RDTs targeting specific malaria proteins were introduced in 1995 [33]. From 2008 to 2015 RDT sales have increased by 182 million units [2], attesting to the popularity of RDTs for a disease where testing in settings with limited infrastructure is common [32].

Three malaria protein biomarkers are commonly targeted by RDTs: *P*. *falciparum* histidine rich protein 2 (*Pf*HRP2), lactate dehydrogenase (LDH) and aldolase [33]. The amino-acid sequence of LDH is conserved across malaria species and “pan-malaria” RDTs detecting the LDH protein can diagnose the presence of *P*. *falciparum*, *P*. *vivax*, *P*. *malariae*, *P*. *ovale* and *P*. *knowlesi* parasites as malaria but do not differentiate between species [33, 34]. LDH was first introduced for malaria diagnostics in 1999 [35] and over 20 monoclonal antibodies with specificity for various *Plasmodium* LDH orthologues have since been developed [34]. Attempts to raise *P*. *knowlesi* specific monoclonal antibodies against LDH have been unsuccessful [36, 37]. Using a combination of the current monoclonal antibodies against LDH, specific diagnosis of *P*. *knowlesi* is possible [34] with good specificity (96%), but unacceptably low sensitivity (32–42, 0–45, 24–73%) [19, 22, 38]. Species-specific epitopes have been identified in the structure of the LDH protein allowing for the detection and differentiation of *P*. *falciparum* and *P*. *vivax* infections [39]. A recently identified new *P*. *falciparum* biomarker, glyceraldehyde-3-phosphate dehydrogenase (GAPDH) has two unique *P*. *falciparum* GAPDH epitopes and an epitope common to GAPDH from all malaria species [40]. Antibodies against each of the GAPDH peptides detected the native protein. A unique epitope in *P*. *knowlesi* GAPDH was identified, but antibody based evidence to support targeting the epitope is not yet available [40].

To date the only definitive diagnosis of *P*. *knowlesi* is by PCR [21], which is more expensive than microscopy or RDTs and requires specialised training and equipment [25, 32]. PCR is considered a reference laboratory conformational tool/test. The first set of nested PCR primers specific for *P*. *knowlesi* detection was developed against the small subunit ribosomal RNA gene [5]. Multiple primer sets and several target genes for nested and real time PCR have since been identified for the detection of *P*. *knowlesi* as reviewed by [25] including a single step PCR target, Pkr140, unique to *P*. *knowlesi*. Loop mediated isothermal amplification methods (LAMP) offer a simpler alternative to PCR but are not as affordable or rapid as RDTs. *P*. *knowlesi* specific LAMP assays target mitochondrial DNA [41], small subunit ribosomal RNA [42], beta tubulin [43, 44], or apical membrane antigen-1 [45] genes.

Currently there are no RDTs or protein biomarkers for the detection of *P*. *knowlesi* infections [8, 21, 22, 44]. This study provides evidence for phosphoethanolamine-N-methyltransferase (PMT) as a malaria biomarker. The PMT gene is absent from the human genome, but present in all 5 human infecting *Plasmodium* species with PMT protein expression confirmed in *P*. *falciparum*, *P*. *vivax* and *P*. *knowlesi* malaria parasites [46–48]. This is a similar characteristic to the popular *P*. *falciparum Pf*HRP2 diagnostic target, but unlike *Pf*HRP2, PMT appears to be essential for parasite development [48–50]. PMT is involved in Plasmodial lipid metabolism and has potential as a drug target [46, 47, 51–55]. Here it is shown that antibodies against the protein or a peptide region of the protein detected Plasmodial PMT and thus could detect a malaria infection. *P*. *knowlesi* PMT and *P*. *falciparum* PMT-specific epitopes were identified and antibodies against these particular epitopes enable the possible identification of *P*. *knowlesi* parasites and differentiation among *P*. *knowlesi*, *P*. *vivax* and *P*. *falciparum* parasites.

## Methods

### Ethics

University of KwaZulu-Natal Animal Ethics Committee approval for the study was obtained. Reference:004/15//Animal. This approval complies with the South African National Standards:SANS 10386:2008, The care and use of animals for scientific purposes, ISBN 978-0-626-22296-3.

### *In silico* and bioinformatics analysis to identify Plasmodial protein and peptide targets

Identification of phosphoethanolamine-N-methyltransferase (PMT) as a potential diagnostic protein target was done *in silico* as described previously [40]. Proteins were ranked based on abundance from transcription and proteomic studies [56, 57]. Peptides that were conserved in all three orthologues (common epitope) or unique to *P*. *falciparum* (*Pf*PMT), *P*. *vivax* (*Pv*PMT) and *P*. *knowlesi* (*Pk*PMT) PMT proteins were selected based on sequence alignment and predicted immunogenicity (Predict7^™^ analyses [58]). The crystal structure of *Pf*PMT was used to assess antibody accessibility of the peptides [55, 59]. The four selected peptide epitopes were synthesized with either N- or C-terminal cysteines for coupling to rabbit albumin and affinity resins (GL Biochem Ltd. Shanghai, China).

### Recombinant expression and affinity purification of three PMT protein orthologues

Plasmids encoding the genes for *P*. *falciparum*, *P*. *vivax*, and *P*. *knowlesi* orthologues of PMT were kindly provided by B. Mamoun (Yale University) [46, 52, 54]. All three PMT orthologues were recombinantly expressed in BL21(DE3) *E*. *coli* (Novagen, Darmstadt, Germany) cells and the sequences of the cloned gene for each protein confirmed by DNA sequencing. The *P*. *falciparum* protein was expressed from a pET 15(b) vector in lysogeny broth (LB) (1% (w/v) tryptone; 0.5% (w/v) yeast extract; 85 mM NaCl; 11 mM glucose) supplemented with 100 μg/ml ampicillin and induced with 1 mM isopropyl thioglucopyranoside (IPTG) for 4 h at 37°C. The other two orthologues were expressed overnight at 37°C from a pET 28(a) vector using auto-inducing terrific broth (1.2% (w/v) tryptone, 2.4% (w/v) yeast extract, 0.4% (w/v) glycerol, 0.231% (w/v) KH_2_PO_4_, 1.254% (w/v) K_2_HPO_4_) [60] supplemented with 25 μg/ml kanamycin. The histidine tagged recombinant proteins were purified on TALON^®^ cobalt affinity resins, according to manufacturer’s instructions as described previously [40]. A 50 mM NaH_2_PO_4_, 300 mM NaCl, 0.02% (w/v) NaN_3_ at pH 8.0 buffer was used throughout with the addition of 10 mM imidazole in the sample loading buffer to reduce the binding of *E*. *coli* proteins to the TALON^®^ affinity resin and 250 mM imidazole in the elution buffer.

### Molecular exclusion chromatography

Four milligrams of the purified recombinant proteins were passed over a molecular exclusion chromatography column (HiPrep 16/60 Sephacryl S-200 column, 120 ml column volume) in 50 mM NaH_2_PO_4_, 150 mM NaCl at pH 8.0 to verify their respective molecular mass. Buffer flow was 0.5 ml/min and the absorbance of eluents monitored at 280 nm and 4 ml fractions collected (ÄKTA Prime Plus, GE Healthcare Life Sciences). The column was calibrated and samples prepared as described previously [40].

### Raising antibodies in chickens against proteins and peptides

Ethical clearance for this study was granted by the animal research ethics committee of the University of KwaZulu-Natal (004/15//Animal) and all institutional guidelines for animal husbandry were adhered to. The chickens used were Hyline Brown, sourced at the University of KwaZulu-Natal, Ukulinga research facility. Animals (layers, 10 weeks old) were fed layers Mash (Meadow feeds, South Africa) *ad libitum* and had constant access to water by means of nipple drip-feed. Animals were euthanased at the end of the experiment by decapitation (AVMA guidelines for the Euthanasia of Animals 2013). Each animal was housed individually and monitored twice daily when eggs were collected and marked. Chickens were used in the experiment because antibodies can be harvested from egg yolks, thus avoiding invasive procedures required for taking blood, fewer animals are required and local inflammatory responses associated with Freund’s complete adjuvant are not seen in chickens [61, 62]. Antigen was emulsified in Freund’s complete adjuvant for the initial immunization only (week 0) and Freund’s incomplete adjuvant for booster immunizations (weeks 2, 4, 6). Animals were immunized in the breast muscle, with 50 μg of the recombinant PMT proteins or 500 μg of the respective peptides conjugated to rabbit albumin used per immunization. Principles recommended by the Hyline Brown Management Guide were adhered to (www.hyline.com/UserDocs/Pages/BRN_COM_ENG.pdf*)*. Antibodies against the whole recombinant proteins or the selected peptides coupled to rabbit albumin carrier were raised and affinity purified as described [39, 63]. Briefly, the isolated IgY was passed over recombinant PMT or peptide affinity columns. Non-specific antibodies were removed by extensive washing and the bound antibodies were then eluted with a change in pH. The resulting polyclonal affinity purified antibodies were used in western blotting and ELISA assays.

### SDS-PAGE and western blotting

Reducing SDS-PAGE gels comprising 4% stacking and 12.5% resolving gels were used throughout [64]. All reference gels were stained with Coomassie Brilliant Blue R-250 [65]. Proteins were transferred electrophoretically to nitrocellulose and the nitrocellulose was blocked with 5% (w/v) low fat milk powder in TBS (20 mM tris; 200 mM NaCl at pH 7.4) for 1 h [66]. Primary chicken IgY or a mouse anti-His_6_ antibody (1:6000) (Merck, Darmstadt, Germany, cat # 05–949) and secondary goat anti-mouse-HRPO (1:10000, cat # 115-035-003), rabbit anti-chicken-HRPO (1:15000, cat # 303-035-003) and rabbit anti-human-HRPO (1:6000, cat # 309-035-003) (Jackson IR laboratories Inc., Baltimore, PA, USA) antibodies were prepared in 0.5% (w/v) BSA-TBS and incubated for 1 or 2 h respectively. All other antibody concentrations are stated in the text. Protein was visualised by developing nitrocellulose bound proteins for 30 min with 3.4 mM 4-chloro-1-naphthol and 0.04% (v/v) H_2_O_2_ as substrate. Enhanced chemiluminescence (ECL) was used to detect native *Pf*PMT in a *Pf*(D10) lysate using a previously described protocol [40]. All images were captured using the G:Box Chemi XR5 system (Syngene).

### Measuring protein concentration

Protein concentrations were determined using the Bradford assay [67]. IgY and human IgG concentrations were calculated using A 280 nm values and the respective extinction coefficients: IgY (ε = 1.25) [68] or human IgG (ε = 1.35) [69].

### Coupling IgY to HRPO

Eight milligrams of horse radish peroxidase (HRPO) (1360 Units, Boehringer Mannheim) was conjugated using the periodate coupling method (omitting the fluorodinitrobenzine reaction) to an equivalent concentration of affinity purified IgY raised against the whole recombinant *Pv*PMT protein.

### ELISA

ELISA plates were coated with sample in PBS overnight at 4°C. Wash steps comprising three PBS-Tween 20 (0.1% (v/v)) and three PBS washes were performed between each incubation step. Wells were blocked with 0.5% (w/v) BSA-PBS (1 h at 37°C) and antibody incubations were performed (1 h at 37°C) in this buffer with Tween 20 (0.1% (v/v)). Either 2,2’-azino-bis(ethylbenzothiazoline-6-sulphonic acid (ABTS) or 3,3’,5,5’-tetramethylbenzidine (TMB) substrate prepared in a 150 mM citrate-phosphate buffer at pH 5.0, was added to the wells and incubated in the dark at room temperature for 1 h. Antibody production in chickens was monitored using an indirect ELISA as described [63]. A double antibody sandwich (DAS) ELISA was optimized using the anti-peptide antibodies as the capture antibodies (between 0.5 to 1 μg) and the anti-r*Pv*PMT-HRPO coupled antibodies (0.5 μg) for detection. For the uninfected whole blood lysate spiked ELISAs, a fresh 1 ml human A-positive blood sample was lysed in 14 ml of 150 mM NH_4_Cl, 10 mM NaHCO_3_, 1 mM Na_2_EDTA, pH 7.4 buffer [70]. Tween 20 was added (0.1% (v/v)) and the lysed blood samples were aliquoted and spiked with the respective recombinant PMT protein orthologues alone or in combinations, or spiked with recombinant *P*. *falciparum* lactate dehydrogenase (r*Pf*LDH control), at 100 ng per ELISA well. Statistical analysis was done using the Student’s t-test, with *p* ≤ 0.05 and ≤ 0.001 indicated with “*” or “**” respectively, where applicable.

### Additivity index

To assess if the antibodies bound the PMT proteins additively or competitively, their respective additivity indices were determined from average absorbance readings in duplicate ELISAs [71]. The recombinant PMT orthologues were coated at 250 ng/well with the following saturating primary antibody concentrations: anti-r*Pf*PMT, anti-*Pv*VYSIKEYNSLKDC (*Pv*PMT peptide), anti-*P*LENNQYTDEGVKC (common PMT peptide) and anti-r*Pk*PMT at 125 ng; anti *Pf*CEVEHKYLHENKE (*Pf*PMT peptide) and anti-r*Pv*PMT at 250 ng; anti-*Pk*LYPTDEYNSLKDC (*Pk*PMT peptide) at 1 μg.

### Human IgG anti-malarial antibodies

Three hundred milligrams of a human anti-malaria antibody pool [72, 73] was used to affinity purify human IgG antibodies against r*Pf*PMT as described for other proteins [74].

## Results

### Recombinant expression, affinity purification and molecular exclusion chromatography of *P*. *falciparum*, *P*. *vivax* and *P*. *knowlesi* phosphoethanolamine-N-methyltransferase (PMT)

**Fig 1:**
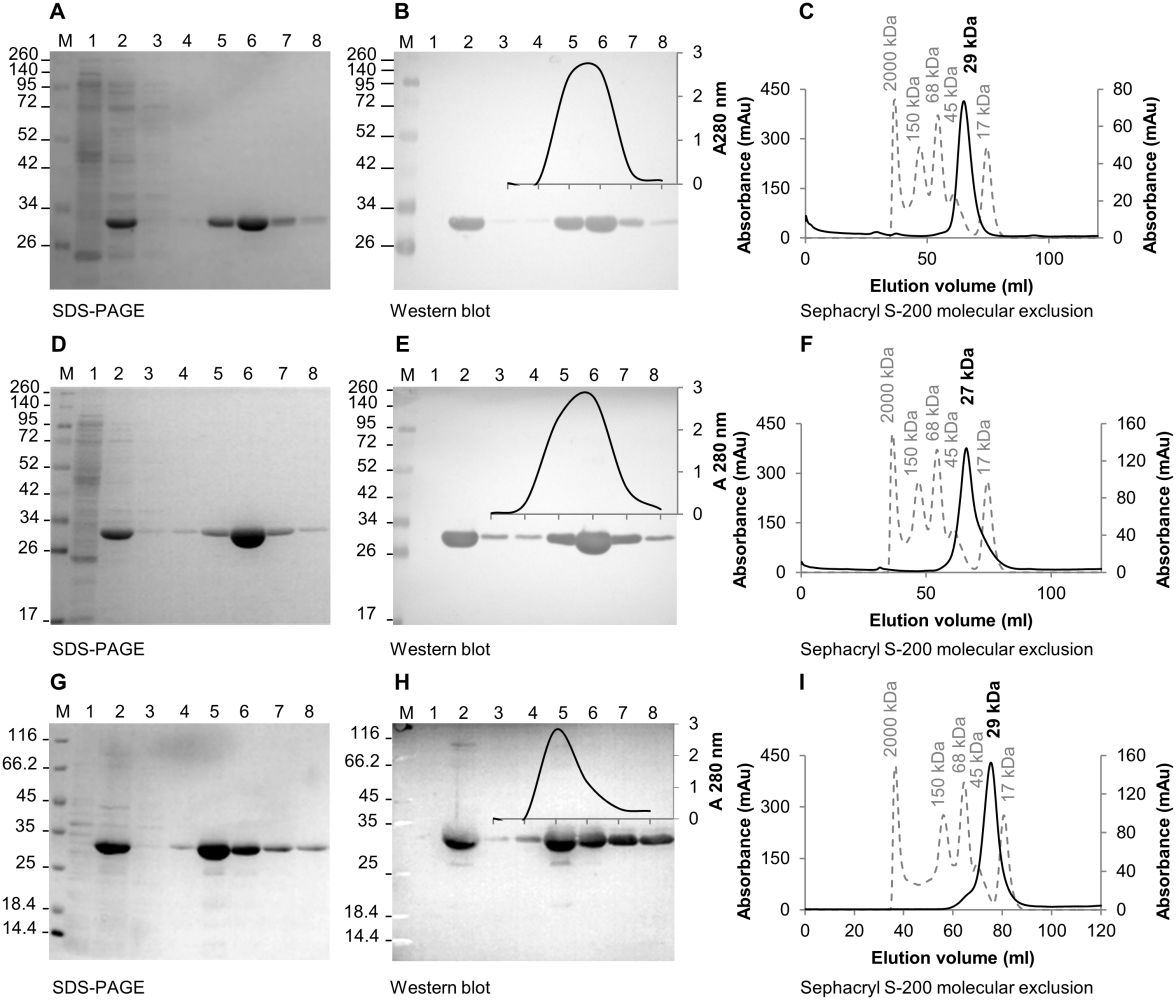
Purification and molecular exclusion chromatography of recombinant *P*. *falciparum*, *P*. *vivax* and *P*. *knowlesi* PMT. Samples (A, D and G) from the respective purification steps of three recombinantly expressed PMT orthologues, purified with an anti-His_6_ affinity resin, were resolved on 12.5% reducing SDS-PAGE gels. Western blots (B, E and H) of the gels were probed with the appropriate antibodies. (M) Molecular weight marker; (lane 1) untransformed *E*. *coli* lysate; (lane 2) induced *E*. *coli* lysate; (lane 3) final wash and (lanes 4 to 8) eluents 1 to 5 from the Talon affinity matrix. The elution profiles (280 nm) were overlaid on the respective blots (B, E and H). Elution profiles (C, F and I) in milli absorbance units (mAu) of each PMT orthologue from a Sephacryl S-200 chromatography matrix. The calibration standards were plotted on the primary axis (dashed line), with the respective sizes indicated above each peak. Each PMT profile (black line) was plotted on the secondary axis and the estimated protein size indicated above the respective peaks in bold.

The orthologues of PMT, from *P*. *falciparum*, *P*. *vivax* and *P*. *knowlesi* were recombinantly expressed in BL21(DE3) *E*. *coli* host cells as histidine tagged proteins and the proteins were affinity purified, using a TALON^®^ resin (Fig 1). Induced *E*. *coli* lysates contained a prominent 29 kDa r*Pf*- and r*Pk*PMT (Fig 1A and 1G, lane 2) and 27 kDa r*Pv*PMT protein band (Fig 1D, lane 2). The same protein bands were detected by an anti-His_6_ tag antibody (Fig 1B, 1E and 1H, lane 2). The intensity of each protein band on the gel corresponded with the A 280 nm elution profile from an affinity matrix (insert in Fig 1B, 1E and 1H, lanes 3–8). No proteins were detected in the untransformed *E*. *coli* lysates (Fig 1B, 1E and 1H, lane 1). The purified recombinant proteins resolved as monomers of 29 kDa for the *P*. *falciparum* and *P*. *knowlesi* proteins and 27 kDa for the *P*. *vivax* protein on a Sephacryl S-200 molecular exclusion chromatography column (Fig 1C, 1F and 1I respectively). The purified recombinant PMT proteins were the source material for raising antibodies in chickens.

### Detection of the recombinantly expressed PMT orthologues with chicken immunoglobulin Y (IgY) raised against PMT from three malaria species

**Fig 2:**
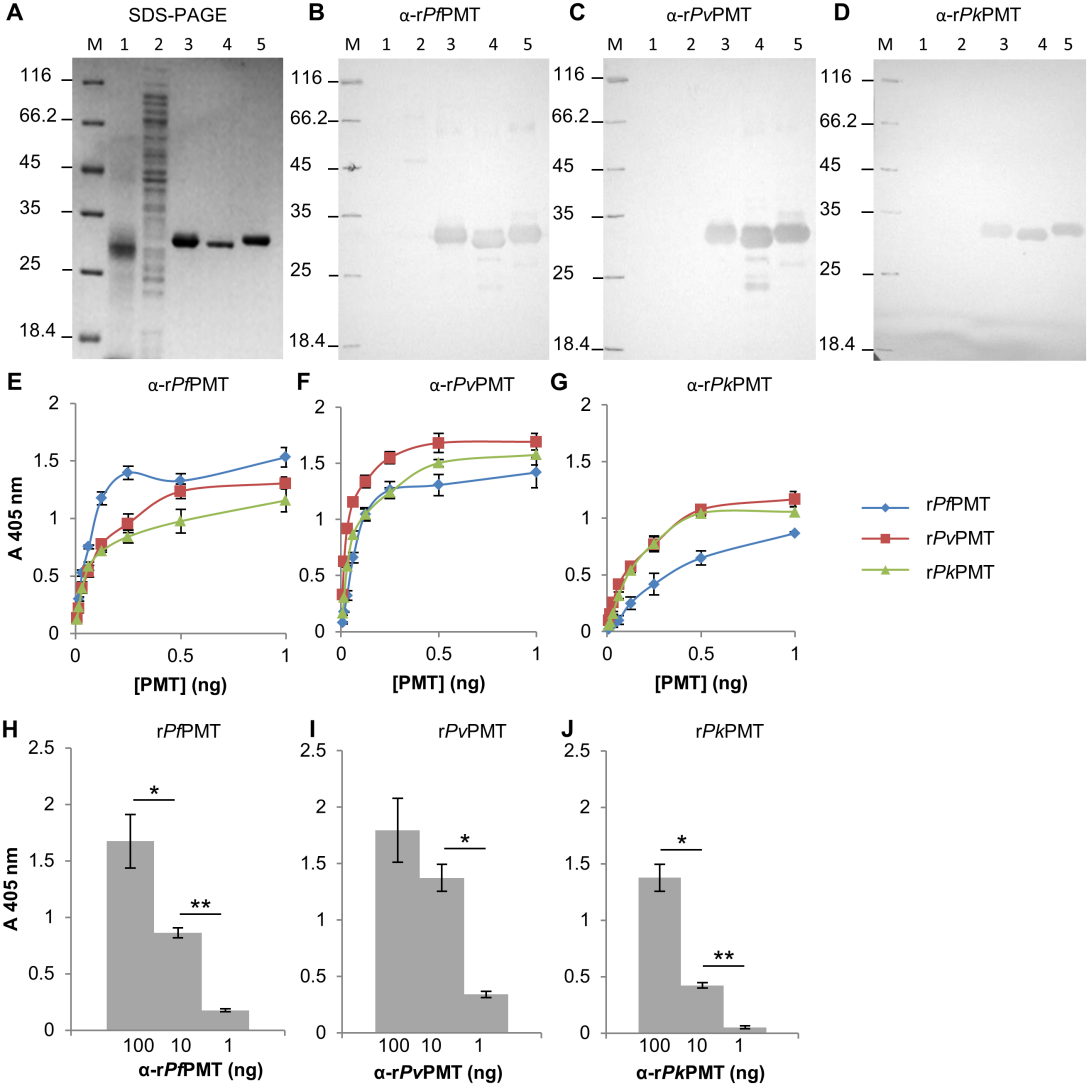
Detection of the recombinant PMT orthologues with the respective anti-recombinant PMT IgY. (A) Reducing 12.5% SDS-PAGE reference gel for (B to D) western blots. The reference gel was the same as shown in Fig 4(A) as all experiments were performed at the same time on the same batch of samples. (M) Molecular weight marker, (lane 1) uninfected red blood cell lysate (25 μg), (lane 2) untransformed *E*. *coli* lysate (25 μg), (lanes 3 to 5) r*Pf*PMT, r*Pv*PMT and r*Pk*PMT respectively. (B) Western blots probed with anti-r*Pf*PMT or (C) anti-r*Pv*PMT or (D) anti-r*Pk*PMT IgY (10 μg) and detected with a rabbit anti-chicken-HRPO secondary antibody. (E to G) IgY against each of the whole recombinant PMT proteins (100 ng) was used to detect a range (0.8 to 100 ng) of concentrations of (E) r*Pf*PMT, (F) r*Pv*PMT and (G) r*Pk*PMT in an ELISA. (H to J) IgY was diluted (100 to 1 ng) and used to detect a single concentration (100 ng) of r*Pf*PMT, r*Pv*PMT and r*Pk*PMT respectively. Antibodies against a protein were denoted as “α” and the protein name. ELISA results present averages of triplicate values with standard deviations. Student’s t-test with *p* ≤ 0.05 and ≤ 0.001 are indicated with “*” or “**” respectively.

Antibodies (IgY) were isolated from the eggs of chickens injected with each of the three PMT proteins and affinity purified using the parent protein coupled to an AminoLink^®^ resin. All the affinity purified antibodies were evaluated in western blots and ELISA and none of the antibodies detected proteins in an uninfected red blood cell lysate or an untransformed *E*. *coli* lysate (Fig 2B to 2D, lanes 1 and 2). Each of the antibodies against each of the PMT proteins from a different species detected the parent protein and both of the other two PMT orthologues in western blots (Fig 2B to 2D, lanes 3 to 5). The size of the protein detected corresponded to that obtained in the SDS-PAGE gels described above. In an ELISA format (Fig 2E to 2G), antibodies against *Pf*PMT and *Pv*PMT had slightly higher reactivity for the parent protein than that for the other orthologues. The anti-r*Pk*PMT antibodies detected the r*Pv*PMT and its r*Pk*PMT parent protein equally. Interestingly the anti-r*Pv*PMT and anti-r*Pk*PMT antibodies detected both r*Pv*PMT and r*Pk*PMT better than the r*Pf*PMT orthologue (Fig 2F and 2G). The lower limits of detection of the antibodies were assessed by titration of the antibodies against a set PMT concentration. The anti-r*Pf*PMT and anti-r*Pv*PMT antibodies detected 1 ng of PMT (Fig 2, 2H and 2I respectively), and the anti-r*Pk*PMT antibodies detected 10 ng of PMT (Fig 2J).

### Selection of a shared or a unique peptide epitope for PMT from each species

**Fig 3:**
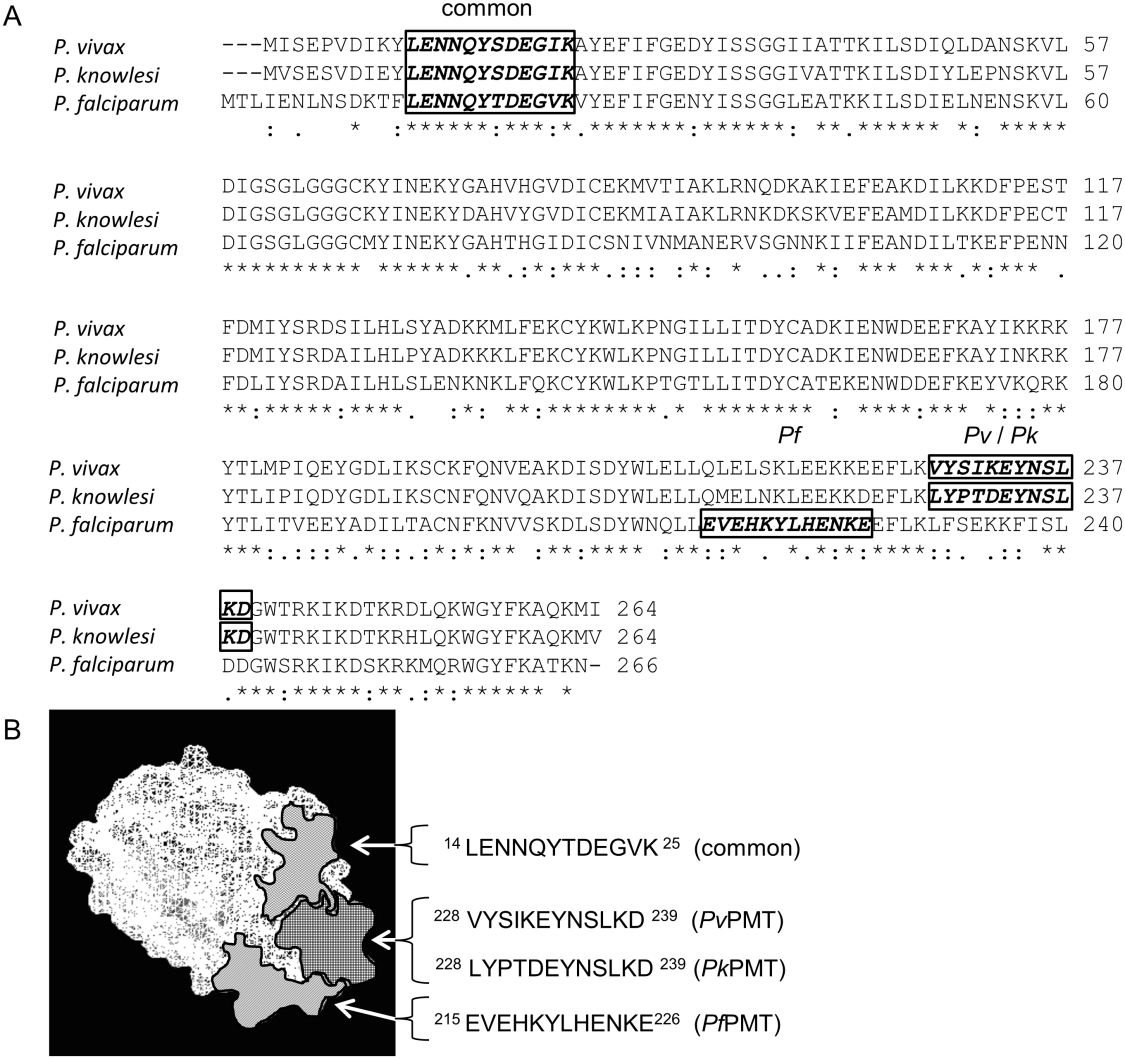
Sequence alignment of PMT orthologues and location of peptide epitopes on the *Pf*PMT crystal structure. (A) Alignment of *P*. *vivax* (XP_001614208.1), *P*. *knowlesi* (XP_002259925.1) and *P*. *falciparum* (Pf3D7_1343000.1) PMT protein orthologues. Potential epitopes selected by Predict7^™^ analysis are indicated on the sequences as the common (boxed “common”) and species specific epitopes (boxed “*Pf*” or “*Pv*” or “*Pk*”). (B) The surface location of each selected peptide epitope is indicated on the 3D crystal structure of *Pf*PMT (3uj6).

Peptides in each PMT protein from a single species were selected as antibody targets. Peptide epitopes were identified by sequence alignment, epitope prediction software (Predict7^™^), 3D crystal structure and BLASTp analyses as described previously (Fig 3; [39, 40]). The four selected peptides are illustrated on an image showing the alignment of the three amino acid sequences (Fig 3A). The image depicts the “common” *P*LENNQYTDEGVKC, the *P*. *falciparum* specific *Pf*CEVEHKYLHENKE, the *P*. *vivax* specific *Pv*VYSIKEYNSLKDC and *P*. *knowlesi* specific *Pk*LYPTDEYNSLKDC peptide sequences. The predicted surface location of each of the peptides was highlighted on a picture of the *Pf*PMT crystal structure (Fig 3B; [55, 59]) showing that the peptide sequences are surface accessible for antibody interaction, are separate and do not overlap.

BLASTp analysis (Table 1) was used to compare the amino acid sequences of the chosen PMT peptides with the corresponding amino-acid sequences found in PMT from other *Plasmodium* species. The BLASTp analysis of the peptides identified Plasmodial PMT sequences with 100% identity and no epitopes with significant sequence identity were found for either human or human pathogen proteins. The peptide epitopes were synthesized, coupled to a rabbit albumin carrier protein and injected into chickens to raise antibodies.

**Table 1:** BLASTp analysis of selected PMT peptide epitopes.

Species	*Pf*PMT^1^	*Pv*PMT	*Pk*PMT	common PMT
***P*. *falciparum***	EVEHKYLHENKE (100)	LFSEKKFISLDD (42)	LFSEKKFISLDD (33)	LENNQYTDEGVK (100)
***P*. *vivax***	QLELSKLEEKKE (42)	VYSIKEYNSLKD (100)	VYSIKEYNSLKD (67)	LENNQYSDEGIK (83)
***P*. *knowlesi***	QMELNKLEEKKD (33)	LYPTDEYNSLKD (67)	LYPTDEYNSLKD (100)	LENNQYSDEGIK (83)
***P*. *ovale***	EMELHRLNEKKE (50)	EYSLKDYNTLKD (67)	EYSLKDYNTLKD (50)	LESYQYSDESIK (58)
***P*. *malariae***	EMEVNRLEQKKE (42)	KYSTKEYESLIN (58)	KYSTKEYESLIN (50)	LENNQYSDEGIK (83)
**Overall identity**	::* *.::*:	:..:: *:	:..:: *:	**. **:**.:*
**BLASTp**	*P*. *falciparum*	*P*. *vivax*	*P*. *knowlesi*	*P*. *falciparum*, *P*. *reichenowi*

^1^. The candidate epitopes were used as BLASTp query sequences to assess their specificity to the *Plasmodium* PMT orthologues and any matches with 100% identity were listed. The amino acid sequences from putative *P*. *ovale* and *P*. *malariae* PMT were included.

### Detection of the recombinantly expressed PMT orthologues with the respective anti-peptide IgY

**Fig 4:**
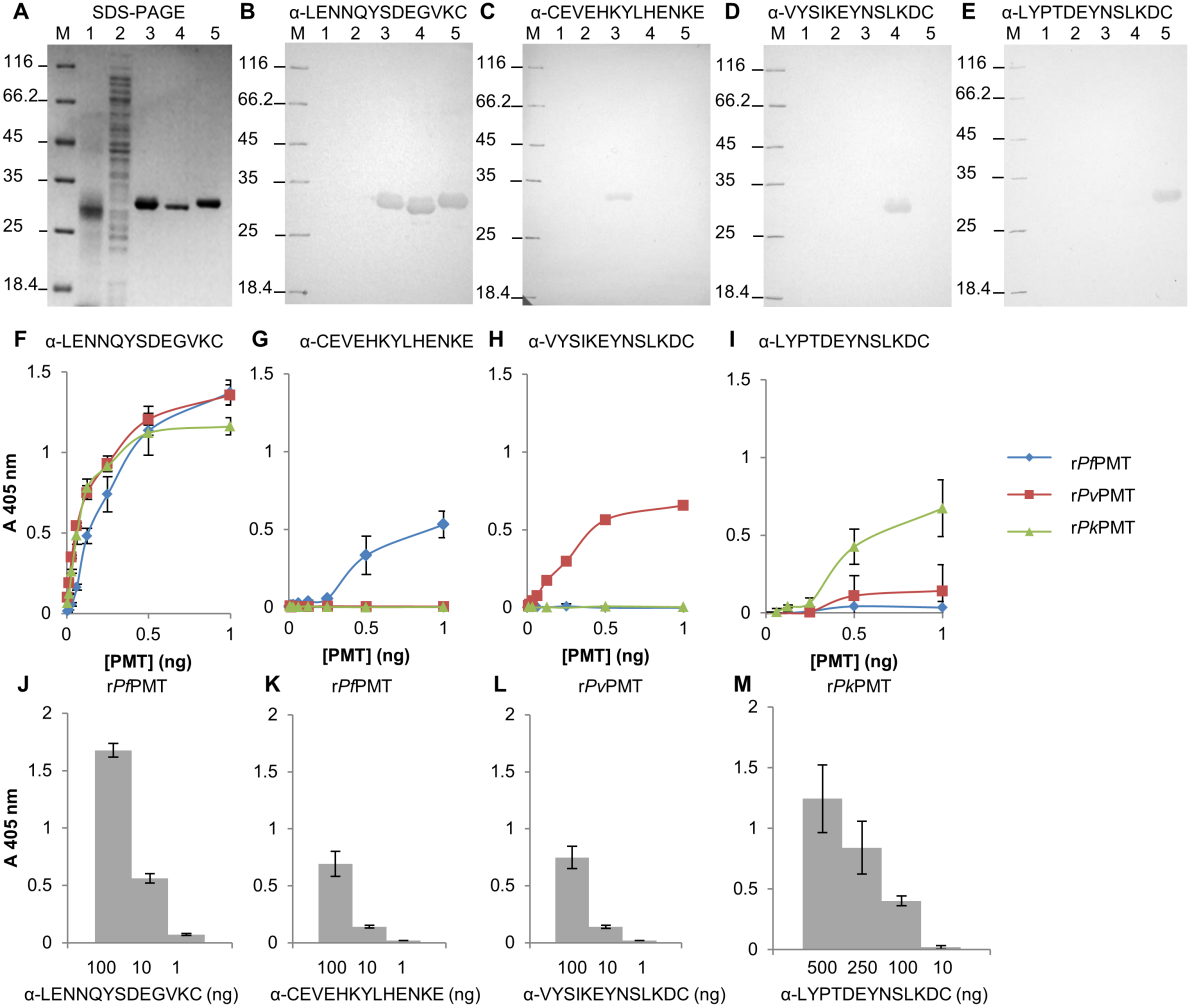
Detection of the recombinant PMT orthologues with the respective anti-PMT peptide IgY. (A) Reducing 12.5% SDS-PAGE reference gel for (B to E) western blots. The reference gel was the same as shown in Fig 2(A). All experiments were performed at the same time on the same samples. (M) Molecular weight marker, (lane 1) uninfected red blood cell lysate (25 μg), (lane 2) untransformed *E*. *coli* lysate (25 μg) and (lanes 3 to 5) r*Pf*PMT, r*Pv*PMT and r*Pk*PMT respectively. (B) Western blots probed with anti-*P*LENNQYTDEGVKC, (C) anti-*Pf*CEVEHKYLHENKE, (D) anti-*Pv*VYSIKEYNSLKDC, or (E) anti-*Pk*LYPTDEYNSLKDC IgY (10 μg) and detected with a rabbit anti-chicken-HRPO secondary antibody using 4-chloro-1-naphthol and H_2_O_2_. (F to I) Detection of r*Pf*PMT, r*Pv*PMT and r*Pk*PMT respectively (0.8 to 100 ng) in an ELISA with anti-peptide IgY (100 ng). (J to M) ELISA plates were coated with the PMT orthologues at 100 ng and detected with different dilutions of anti-PMT peptide IgY, with 100 to 1 ng (J to L) and 500 to 10 ng (M). All ELISAs were done in triplicate and the standard deviations included. Antibodies against a peptide were denoted as “α” and the peptide sequence. Student’s t-test with *p* ≤ 0.05 and ≤ 0.001 are indicated with “*” or “**” respectively.

Antibodies raised against each peptide in chickens were affinity purified using the respective parent peptides coupled to a SulfoLink^®^ affinity resin and evaluated in a western blot and a direct ELISA (Fig 4). None of the anti-peptide antibodies detected any proteins in either an uninfected red blood cell lysate, or an untransformed *E*. *coli* lysate (Fig 4B to 4E, lanes 1 and 2). The antibodies against the common peptide (*P*LENNQYTDEGVKC) detected all three PMT orthologues at the appropriate sizes in a western blot (Fig 4B, lanes 3 to 5) and with similar intensity of signal in an ELISA format (Fig 4F). The antibodies against the *P*. *falciparum* PMT unique peptide (*Pf*CEVEHKYLHENKE), the *P*. *vivax* (*Pv*VYSIKEYNSLKDC) or *P*. *knowlesi* (*Pk*LYPTDEYNSLKDC) peptides only detected their respective PMT proteins from the parent species in a western blot (Fig 4C, lane 3, D and E, lanes 4 and 5 respectively) and an ELISA (Fig 4G, 4H and 4I respectively). The strongest signals were obtained for antibodies against the common peptide (Fig 4J to 4M). The limit of detection of the anti-peptide antibodies was assessed by titration of the antibodies against a set series of PMT concentrations. The anti- *P*. *falciparum* (*Pf*CEVEHKYLHENKE) and *P*. *vivax* (*Pv*VYSIKEYNSLKDC) specific epitope antibodies detected their specific PMT protein orthologues at similar concentrations (10 ng; Fig 4K and 4L respectively), but the anti- *P*. *knowlesi* peptide (*Pk*LYPTDEYNSLKDC) antibodies detected r*Pk*PMT at a higher concentration (100 ng; Fig 4M).

### Evaluating epitopes as antibody targets for the capture and detection of PMT in an ELISA

To develop an ELISA or RDT where both a capture and a detection antibody against the same protein are required, it is necessary to evaluate potential competition in binding to the protein between the two antibodies. This was done by evaluating the additivity of the different antibody combinations (anti-peptide and anti-whole protein antibodies) in ELISA formats. If the signal increases with the addition of the second antibody there is little competition. All the whole protein and anti-peptide antibodies generated a signal that increased by 50% or more indicative of a lack of competition (Table 2). Additivity values above 70% were obtained for 5 of the combinations and the highest (100%) were obtained with the anti-*Pv*VYSIKEYNSLKDC anti-*P*LENNQYTDEGVKC combination; the anti-r*Pk*PMT whole protein anti-*Pk*LYPTDEYNSLKDC combination and the anti-*Pk*LYPTDEYNSLKDC and anti-*P*LENNQYTDEGVKC combination (Table 2).

**Table 2:** Additivity indices of antibodies raised against the recombinant PMT orthologues and the selected peptide epitopes.

Antibody	α-*Pf*CEVEHKYLHENKE	α-*P*LENNQYTDEGVKC
α-r*Pf*PMT	55.9*	59.5
α-*Pf*CEVEHKYLHENKE	NA	83
Antibody	α-*Pv*VYSIKEYNSLKDC	α-*P*LENNQYTDEGVKC
α-r*Pv*PMT	83.3	61.8
α-*Pv*VYSIKEYNSLKDC	NA	100
Antibody	α- *Pk*LYPTDEYNSLKDC	α-*P*LENNQYTDEGVKC
α-r*Pk*PMT	100	71.7
α-*Pk*LYPTDEYNSLKDC	NA	100

*All additivity index values represent increased signal percentages calculated from average absorbance values.

**Fig 5:**
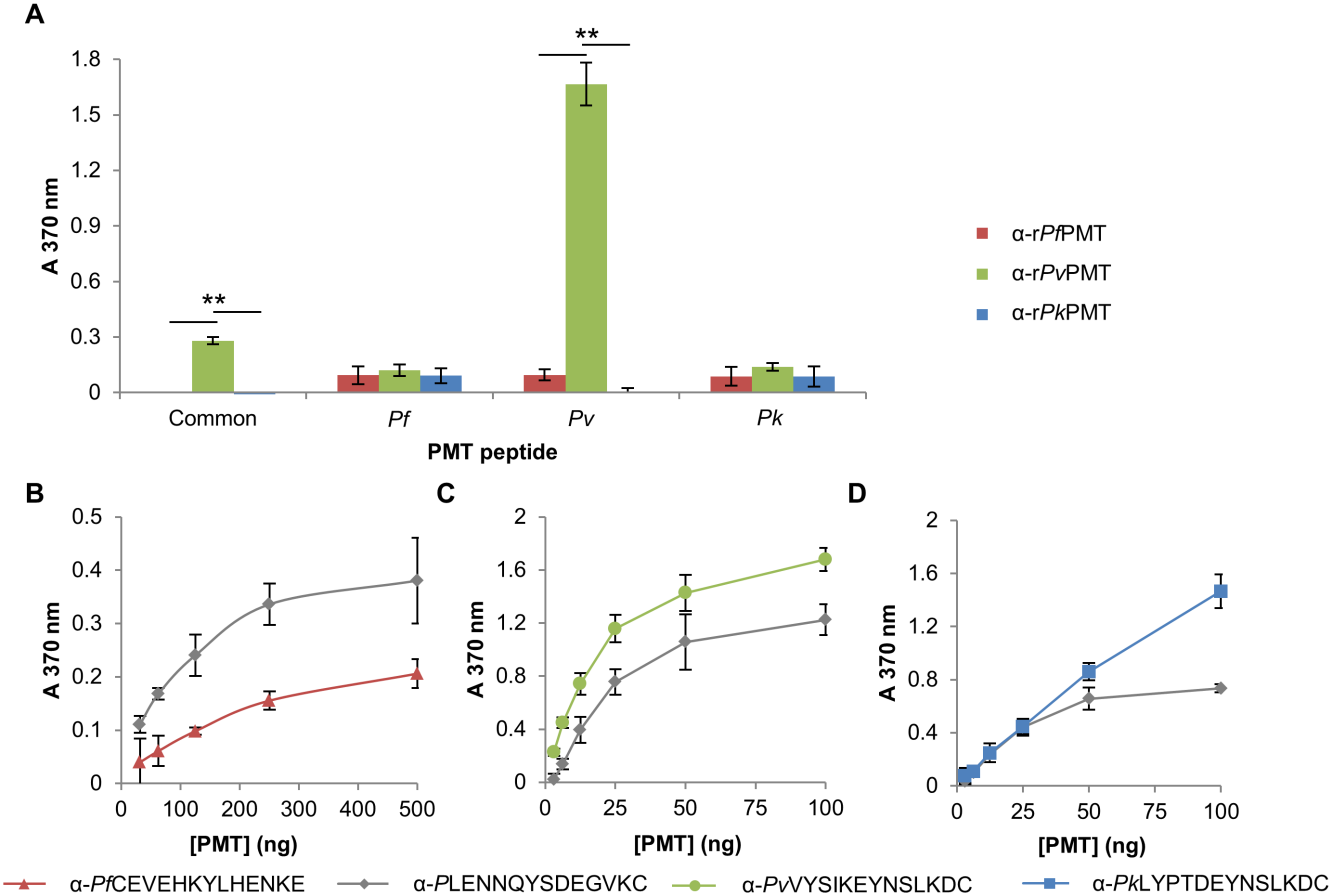
PMT peptide detection with the anti-rPMT antibodies and DAS-ELISA based capture of rPMT orthologues. (A) PMT peptides coated at 100 ng were detected with the anti-recombinant PMT IgY (100 ng). (B to D) The recombinant PMT proteins were captured with the anti-peptide antibodies and detected with the anti-PvPMT-HRPO conjugate. All results represent triplicate values with standard deviations. Antibodies against a peptide were denoted as “α” and the peptide sequence. Student’s t-test with *p* ≤ 0.05 and ≤ 0.001 are indicated with “*” or “**” respectively. Note the different scale in (B) compared to (C and D).

It was important to evaluate the peptide epitopes as possible antibody targets for the capture and detection of the PMT protein (Fig 5). The IgY antibodies raised against the whole recombinant PMT orthologues were tested for their reactivity with the selected peptides (Fig 5A). Only the anti-r*Pv*PMT IgY reacted strongly with the *P*. *vivax* specific (*Pv*VYSIKEYNSLKDC) and all other interactions were negligible (Fig 5A). When combining the antibodies in a capture and detection ELISA (Fig 5B, 5C and 5D), using the antibodies against the common peptide as the capture antibody, resulted in lower detection signals compared to using the species specific anti-peptide antibodies, with r*Pf*PMT being the only exception. This was in agreement with the additivity results (Table 2). Antibodies or anti-peptide antibodies against r*Pf*PMT produced the lowest signals in the ELISA tests in comparison to the ELISAs detecting the two other PMT orthologues.

### Detecting PMT in spiked uninfected human blood lysates and native PMT in a *Pf*(D10) culture lysate by DAS ELISA

**Fig 6:**
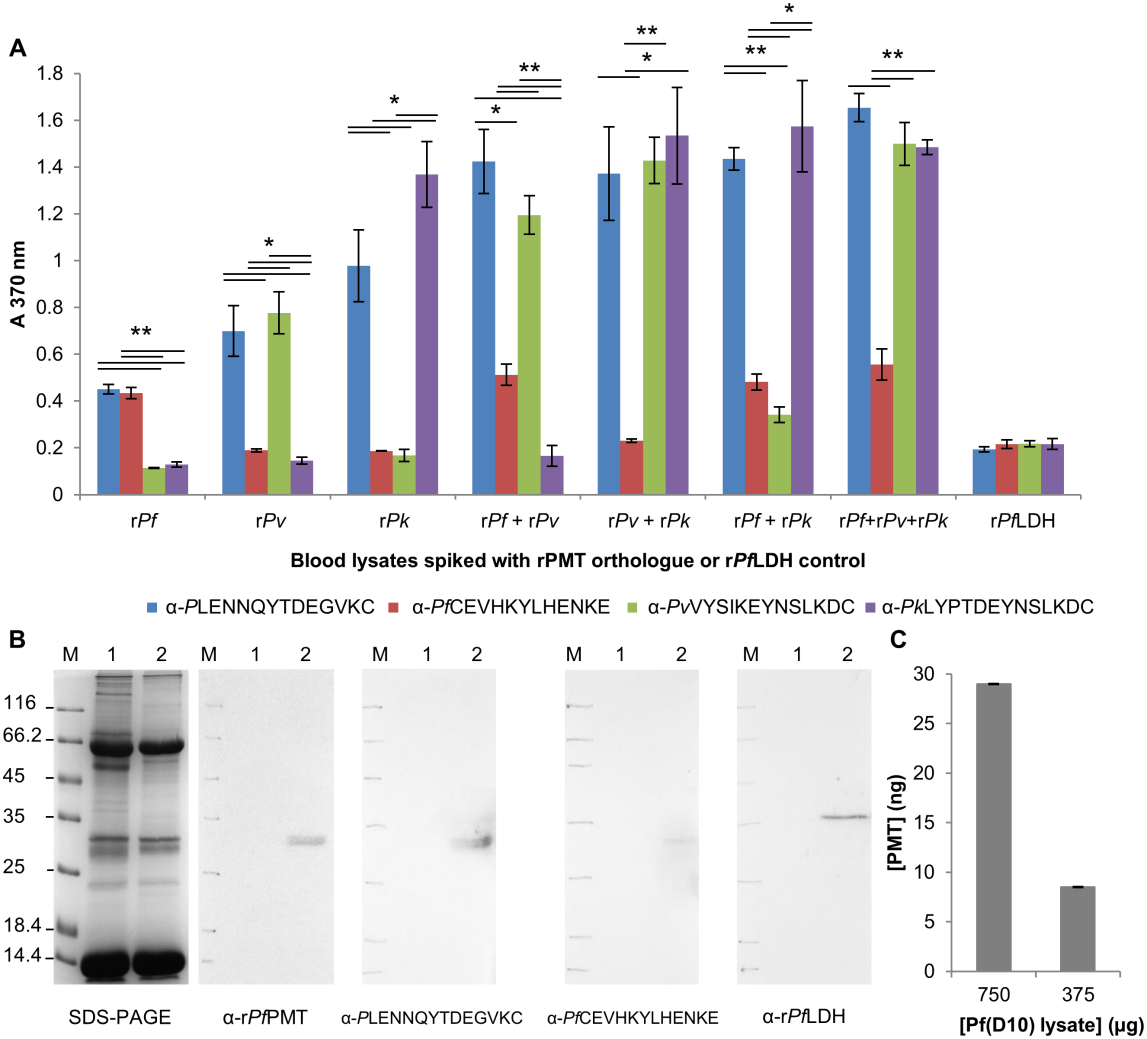
Spiked blood DAS-ELISAs and detection of native *Pf*PMT in a *Pf*(D10) parasite culture lysate. (A) Uninfected, A-positive human whole blood lysates were spiked with recombinant PMT orthologues (100 ng) and captured with anti-peptide IgY (all coated at 500 ng, except anti-*Pf*CEVEHKYLHENKE at 1 μg). Captured PMT proteins were detected with anti-r*Pv*PMT-HRPO coupled IgY (500 ng). r*Pf*LDH (100 ng) spiked blood lysates served as a negative control. (B) Native *P*. *falciparum* PMT was detected on western blots, with the Coomassie stained reference gel on the left (SDS-PAGE). (M) Molecular weight marker, (lane 1) uninfected A-positive human whole blood lysate (100 μg) and (lane 2) *Pf*(D10) culture lysate (100 μg). Western blots were probed with: anti-r*Pf*PMT (10 μg); anti-*P*LENNQYTDEGVKC or anti-*Pf*CEVEHKYLHENKE or anti-r*Pf*LDH IgY (100 μg). (C) Detection of native *P*. *falciparum* PMT in a *Pf*(D10) culture lysate with the anti-*Pf*CEVEHKYLHENKE as capture and anti-r*Pv*PMT-HRPO as the detection antibody. TMB + H_2_O_2_ was used as the substrate in all ELISAs, each performed in triplicate with standard deviations shown. Antibodies were denoted as “α” and the peptide sequence or protein name. Student’s t-test with *p* ≤ 0.05 and ≤ 0.001 are indicated with “*” or “**” respectively.

During the red blood cell cycle, as the parasites rupture and invade new host red cells, parasite proteins, like LDH and *Pf*HRP2 are released into peripheral circulation and can be detected to diagnose infection. Therefore any potential interference by blood cell proteins when detecting the PMT protein was evaluated. In an ELISA, PMT proteins were detected by all the antibodies and blood cell lysate components did not influence the assay (Fig 6). Antibodies raised against the common epitope captured all three PMT orthologues from the spiked blood samples. The anti-peptide antibodies against PMT from each species detected only the parent proteins in the spiked blood cell lysates (Fig 6A). When blood was spiked with recombinant *Pf*PMT and *Pv*PMT there was a high signal for the anti-*Pv*PMT peptide antibody. This signal was lower than that for the anti-*Pf*PMT antibody (P<0.05). The anti-*Pf*PMT peptide antibody had the lowest signal of all the anti-peptide antibody combinations in the ELISA. Native *Pf*PMT was detected as a 29 kDa protein in a *Pf*(D10) infected culture lysate by western blot (Fig 6B) with the anti-r*Pf*PMT IgY as well as the antibodies raised against the common (*P*LENNQYTDEGVKC) and *P*. *falciparum* specific (*Pf*CEVEHKYLHENKE) peptides. In the ELISA format *Pf*PMT was captured using the anti-*Pf*CEVEHKYLHENKE antibodies (Fig 6C) and detected approximately 28 ng in the 750 μg of *Pf*(D10) lysate protein.

### Presence of anti-*Pf*PMT antibodies in a human anti-malaria antibody pool

A human anti-malaria antibody pool [72, 73] was passed consecutively over four affinity resins with recombinant *P*. *falciparum* LDH, GAPDH, Cox17 and PMT coupled to the resins. Approximately 0.41 mg of affinity purified human antibodies detecting r*Pf*PMT was eluted from the r*Pf*PMT affinity resin (Table 3). The yield of antibodies against r*Pf*PMT was lower than those that bound to recombinant *P*. *falciparum* LDH and similar (0.42 mg) to those binding recombinant *P*. *falciparum* GAPDH ([40]) on an affinity matrix. The lowest yield was from antibodies binding to recombinant *P*. *falciparum* Cox17, a copper chaperone [74].

**Table 3:** Yields of human IgG against four recombinant *P*. *falciparum* proteins.

Target protein	Affinity pure human IgG (mg)
r*Pf*PMT	0.41
r*Pf*LDH	0.87*
r*Pf*GAPDH	0.42*
r*Pf*Cox17	0.31*

*The human antibody yields against r*Pf*LDH, r*Pf*GAPDH [40] and the r*Pf*Cox17 [74] have been reported previously.

**Fig 7:**
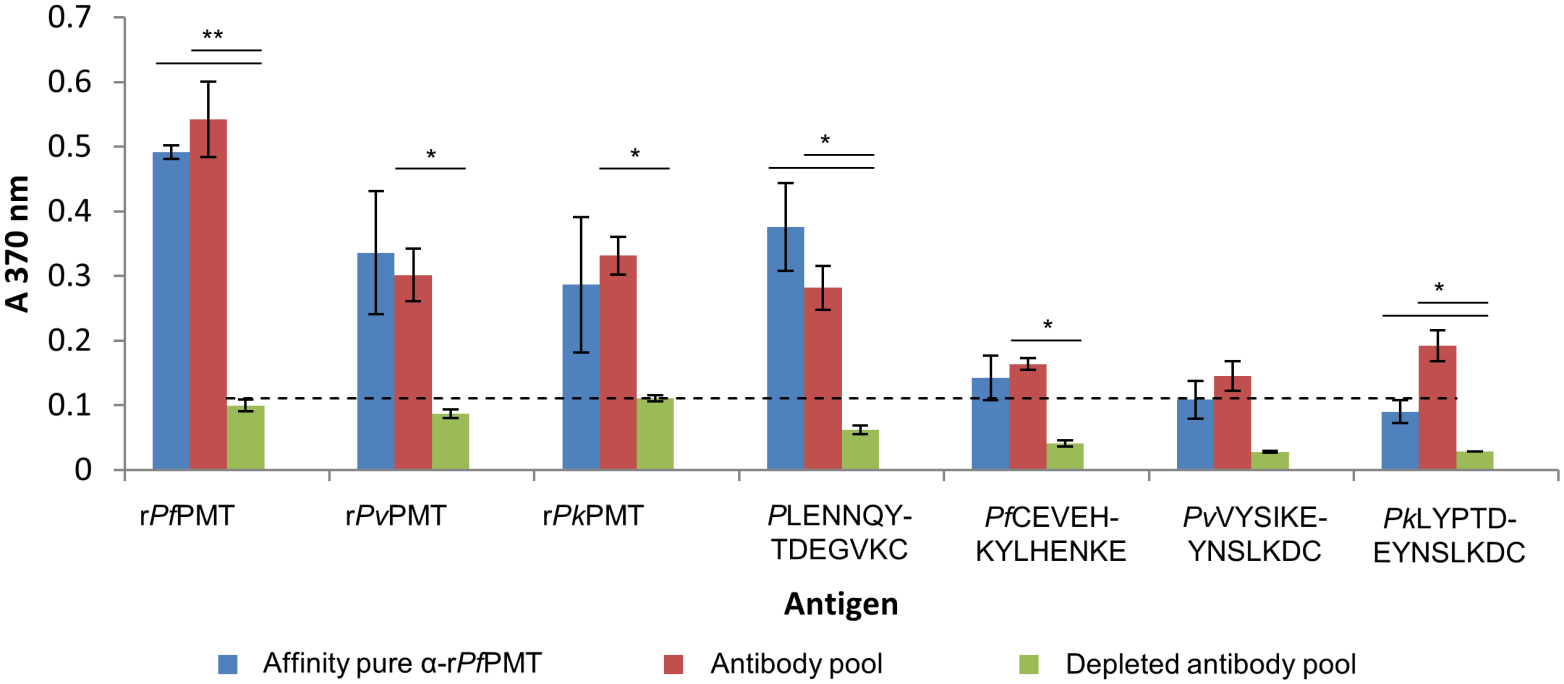
Detection of anti-r*Pf*PMT antibodies in a human anti-malaria hyperimmune antibody pool. A human anti-malaria hyperimmune antibody pool was passed over a r*Pf*PMT affinity column. The affinity purified anti-r*Pf*PMT human antibodies (100 ng) were used to detect each of the recombinant PMT orthologues or each of the PMT peptide epitopes coated directly onto ELISA plates at 100 ng per well. The pool before (antibody pool) and the pool after (depleted antibody pool) passing the human antibodies over the r*Pf*PMT affinity resin were used at 10 μg. All readings were done in triplicate with standard deviations shown. A cut-off value for positive reactions of three times the standard deviation of the background control was included as a horizontal dashed line. Student’s t-test with *p* ≤ 0.05 and ≤ 0.001 are indicated with “*” or “**” respectively.

The human antibodies affinity purified on the r*Pf*PMT affinity matrix detected the PMT protein in an ELISA format (Fig 7), and the limit of detection was 100-fold higher than the crude human antibody pool before isolation of the antibody. The unbound human antibodies from the r*Pf*PMT affinity resin did not react with any of the proteins tested at the same concentration. Both the crude and r*Pf*PMT specific human antibodies reacted with both the *P*. *vivax* and the *P*. *knowlesi* PMT orthologues as predicted from the similarity in amino acid sequence between the proteins and from data with IgY raised against the whole recombinant PMT orthologues (Fig 2B to 2D and 2E to 2G). Interestingly the human antibodies detected the common peptide (*P*LENNQYTDEGVKC), (Fig 7), unlike the IgY (Fig 5A). Reactivity with the other PMT peptides was negligible compared to the background control.

## Discussion

A *P*. *knowlesi* infection is difficult to diagnose with microscopy [25, 26] and can be diagnosed with PCR [25] or LAMP assays [41–45]. A specific point-of-care rapid diagnostic test to detect a *P*. *knowlesi* infection is not available at present [8, 21, 22, 44]. Such a test would improve diagnosis and treatment of the infection and aid in tracking the spread of this species in the Southeast Asia region. We identified phosphoethanolamine-N-methyltransferase (PMT) as a *P*. *knowlesi* specific protein biomarker with the potential to identify a *P*. *knowlesi* infection and to differentiate *P*. *knowlesi* from a *P*. *falciparum* or *P*. *vivax* infection.

### PMT selection and background to the protein

The Plasmodial database [75] was screened to identify proteins that are highly expressed, are expressed by the parasite and not the host or have unique peptide sequences, and can be recombinantly expressed as soluble proteins [40]. Based on these parameters PMT was identified as an attractive target. The enzyme is expressed by *P*. *falciparum*, *P*. *vivax* and *P*. *knowlesi* species and is not expressed by the host [46–48]. Based on *in silico* data, the protein is expressed at similar or higher levels than the current diagnostic target, LDH [40, 56, 57]. An additional advantage is that the protein, unlike *Pf*HRP2, the most popular protein diagnostic target, has been shown to be essential for the parasite in different stages of development [48–50]. The implication is that the gene for the protein is unlikely to be deleted from the parasite’s genome as has been recorded for the *Pf*HRP2 gene in a number of geographic locations [76–79]. PMT has been characterised and is being explored as an antimalarial drug target [46, 47, 51–55].

### Recombinant PMT orthologue model

The PMT gene is present and the protein is expressed by *P*. *falciparum*, *P*. *vivax* and *P*. *knowlesi* parasites [46, 54]. The gene is also present in *P*. *ovale* and *P*. *malariae* genomes ([75], NCBI accessed Aug. 2017). PMT appears to be expressed in *P*. *reichenowi* and *P*. *gallinaceum*, but is absent from all rodent malaria species [53]. The affinity purified recombinant PMT proteins resolved on a SDS-PAGE gel and in gel chromatography at 29 kDa for the *P*. *falciparum* and *P*. *knowlesi* proteins and a 27 kDa *P*. *vivax* protein as predicted from the gene sequences (Fig 1; [46, 52, 54, 75]). The closest human homolog to PMT is a histamine methyltransferase, which is also a S-adenocyl-L-methionine dependent methyl transferase with 7–16% sequence identity and 31% sequence similarity around the substrate binding site [80]. This human protein has insufficient amino-acid identity to be detected by antibodies raised against the malarial proteins. A human pathogen, *Cyclospora cayetanensis* protein shared 39% identity overall. Affinity purified chicken antibodies against each of the three PMTs detected all three orthologues at the appropriate molecular masses in western blots but did not detect uninfected human red blood cell proteins (Figs 2 and 6). This result confirms the purity of the immunisation material and suggests that the antibodies have potential in a diagnostic test.

### Peptide selection

Antibodies against peptide epitopes within the amino acid sequences of the *P*. *falciparum* HRP2 and LDH have been used to identify both the parent protein and used to detect malaria infections [81, 82]. Anti-peptide antibodies against unique peptides in the amino acid sequence of malaria LDH or GAPDH were shown to differentiate between the proteins [39, 40]. An antibody against a common peptide present in the amino acid sequence of orthologues of the malaria specific protein would enable the detection of the protein in all species expressing the protein and by implication detect an infection by any of those species. Similarly antibodies against a peptide unique to a *P*. *knowlesi* protein sequence would enable identification of a *P*. *knowlesi* infection. Anti-peptide antibodies raised against both common and species specific peptide motifs within the amino acid sequence of the PMT protein detected the parental proteins (Fig 3 and Table 1). The species-specific antibodies detected only their parental PMT protein and differentiated between the PMT protein from each species, as predicted by sequence alignment and found for LDH and GAPDH peptides (Fig 4 and Table 1; [39, 40]). PMT malaria orthologues share 61 to 88% sequence identity, which is lower than that described for malarial LDH [54]. Since nonsynonymous sequence mutations on *Pf*HRP2 have had detrimental effects on *Pf*HRP2 based RDTs the potential presence of mutations in PMT sequences was evaluated [76–79]. Alignment of the PMT amino acid sequences from all available isolates of the same species showed a single I192T mutation which lies outside of any of the chosen peptide sequences. The *P*. *vivax* and *P*. *knowlesi* isolates sequenced to date appear not to have any mutations in the sequences of the PMT genes (accessed August 2017). The essentiality of the gene for the parasite, coupled with the lack of mutations found in gene sequences available to date, support the potential of PMT as a diagnostic reagent.

### Antibody characterisation and compatibility

Antibodies raised against peptides and the whole protein were evaluated for use in an antigen capture and detection ELISA (Table 2). Two antibodies detecting the same protein target molecule may either bind competitively (with minimal additivity) or additively i.e. combine to increase the signal [71]. The antibodies raised against the whole proteins combined with the anti-peptide antibodies had additivity indices above 50%, suggesting all the combinations of antibodies could be used in an ELISA or RDT format [83]. Combining the species specific antibodies with anti-common peptide antibodies gave the highest additivity suggesting these to be the best combinations for capture and detection of the PMT protein (Figs 5 and 6). Antibodies against each whole protein detected all the orthologues. The anti-r*Pv*PMT and anti-r*Pk*PMT antibodies detected both r*Pv*PMT and r*Pk*PMT better than the r*Pf*PMT orthologue. This is thought to be due to 88% sequence identity shared between *Pv*PMT and *Pk*PMT in comparison to 64 and 62% shared with *Pf*PMT respectively [54]. Interestingly only the anti-r*Pv*PMT antibodies detected the corresponding specific species epitope in a direct ELISA (Fig 6), which suggests that the other epitopes are not immunogenic in chickens when displayed on the protein in the context of the whole amino-acid sequence.

### Capture and detection of PMT in solution

Antibodies against the PMT protein from each of the three species, detected their partner protein in an ELISA and there was no interference when a blood lysate was added to the assay (Fig 6). Each of the anti-peptide antibodies bound to the respective PMT protein harbouring the appropriate peptide, including in a blood lysate spiked with combinations of the PMT orthologues. Antibodies against the common PMT peptide (*P*LENNQYTDEGVKC), detected all three *P*. *falciparum*, *P*. *vivax* and *P*. *knowlesi* PMT proteins in a spiked blood lysate. Antibodies against the *P*. *falciparum* PMT protein or the common epitope or the *P*. *falciparum* epitope detected PMT in a *P*. *falciparum* infected lysate (Fig 6). The antibody against the *Pf*PMT peptide produced the lowest signal in the ELISA (Fig 6A) where it was marginally better than the anti-*Pv*PMT peptide antibody and gave a poor signal in the western blot (Fig 6B). We are looking for a better *Pf*-specific PMT peptide antibody combination. The antibodies used here were raised in chickens. Chicken IgY does not cross-react with human rheumatoid factor and chicken antibodies are stable at 4°C for long periods indicating their diagnostic potential [84]. A combination of RDTs detecting LDH could be used to detect *P*. *knowlesi* infections [34] albeit with unacceptably low sensitivity [19, 22, 38]. As *P*. *knowlesi* parasitemia during infection increases so rapidly compared to *P*. *falciparum*, it is important to diagnose the correct species as soon as possible. At present this cannot be done with microscopy or RDTs, but can be done with PCR based methods [21]. The PMT protein is suggested as a candidate protein for evaluation in rapid diagnostic tests.

PMT was detected at 28 ng per 1 ml of a 1% *Pf*(D10) culture lysate, which is a similar concentration to *Pf*LDH in the same lysate sample (Fig 6; [40, 85]) and corresponds to a ranking of protein abundance based on mRNA and expression data [40, 86]. This concentration is about five to six times lower than GAPDH (139 ng/ml; [40]) or *Pf*HRP2 (164.5 ng/ml; [85]). In culture *Pf*PMT expression increased by three-fold as the parasite progressed from ring to trophozoite stages [46, 57, 87]. The protein is also expressed in gametocyte [48] and sporozoite stages of the life cycle [75]. The presence of the PMT protein in all stages of parasite development, like LDH, supports its potential for diagnosis. The antibodies raised here are predicted to detect the protein in gametocytes and sporozoites, though this has not yet been evaluated.

PMT is involved in the synthesis of the major membrane phospholipid, phosphatidylcholine and is a soluble protein that localises to the Golgi apparatus [46, 87, 88]. Interestingly PMT mRNA transcript and protein levels are carefully regulated by the parasite, dependent on the choline concentration and membrane biogenesis [88]. The predicted half-lives of *Pf*LDH, *Pf*PMT and *Pf*HRP2 proteins based on amino acid sequences are similar, however the half-life determined *in vivo* of *Pf*LDH and *Pf*HRP2 are very different [89, 90]. Proteins with short half-lives, like *Pf*LDH are more accurate indicators of current infections and useful for tracking disease progress and treatment success and it would be interesting to evaluate the half-life of PMT *in vivo* for this reason.

### Human anti-malaria antibody pool

Circulating host antibodies against malarial proteins have been suggested to interfere with *Pf*HRP2 based RDTs, while LDH based tests appear to be unaffected [91]. A pool of human anti-malaria antibodies from 800 donors was screened for the presence of antibodies against several *P*. *falciparum* proteins (Table 3; [40, 74]). The human antibodies, like those raised in chickens against the whole PMT proteins, detected PMT from all three malaria species (Figs 2 and 7). Interestingly the common epitope was the only peptide of the three detected by this pool of human antibodies and differs from the epitope detected by IgY suggesting that either the recombinant protein assumes a different conformation to that of the native protein, or the response to native protein in humans differs to a response generated in the presence of adjuvant in chickens (Figs 7 and 6 respectively). Since the recombinant protein has been shown to have enzyme activity, it is thought that the latter explanation is the more likely [45 – 48]. The detection of the recombinant protein by the human antibody pool indicates that the recombinant protein and the native protein share structural features. The low concentration of anti-PMT antibodies in the pool, which are lower than anti-*Pf*LDH antibodies, suggests that circulating antibodies in the host are unlikely to reduce the efficacy of a RDT using PMT as the target antigen. Several LDH based diagnostic tests detect parasite LDH despite the presence of antibodies against *P*LDH in human serum [39, 88].

### Peptide epitope comparisons

A *P*. *vivax* specific LDH epitope was identified and antibodies against the peptide differentiated between *P*. *vivax* and *P*. *falciparum* LDH protein [38]. The *P*. *vivax* LDH specific peptide, has been found with further analysis, to share 92% sequence identity with *P*. *knowlesi* LDH. The antibodies against the *P*. *vivax* peptide are likely to detect a *P*. *knowlesi* infection and enable differentiation between a *P*. *knowlesi* and a *P*. *falciparum* infection, but not between *P*. *vivax* and *P*. *knowlesi* infections. In a similar manner a GAPDH peptide was identified that could be a target for anti-peptide antibodies to differentiate between *P*. *falciparum* and *P*. *knowlesi* GAPDH [39]. The *P*. *knowlesi* GAPDH peptide shares 79% sequence identity with the *P*. *vivax* GAPDH sequence which may be sufficient to generate a *P*. *knowlesi* specific anti-peptide antibody. Given that *P*. *vivax* and *P*. *knowlesi* share a common ancestry and are predicted to be evolutionarily closer to each other than to *P*. *falciparum*, it is likely that identifying proteins as targets to differentiate between *P*. *vivax* and *P*. *knowlesi* infections may prove difficult as shown above for the LDH and GAPDH sequences. In this context, the description of a unique *P*. *knowlesi* epitope in the PMT protein sequence strengthens the importance of the PMT protein as a diagnostic candidate. The recommendation in *P*. *knowlesi* endemic regions is that microscopic identification of *P*. *malariae* be diagnosed as *P*. *knowlesi*/*P*. *malariae* [26]. Based on the amino acid sequences of the *P*. *knowlesi* and *P*. *malariae* PMT proteins, antibodies against the *P*. *knowlesi* specific peptide will not detect the *P*. *malariae* PMT protein. Conversely there is a unique region in the *P*. *malariae* amino acid sequence (within the same region on the protein as the *P*. *knowlesi* specific peptide) that could be used to raise antibodies to detect *P*. *malariae* PMT and hence a *P*. *malariae* infection. At present we do not have *P*. *malariae* genomic DNA or the recombinant *P*. *malariae* PMT protein to evaluate this possibility. Given the presence of species-specific epitopes able to generate polyclonal antibodies in chickens in this study, it is predicted that either mouse monoclonal antibodies or phage expressed single chain variable fragment (scFv) antibodies can be raised/selected to detect *P*. *knowlesi* PMT and diagnose a *P*. *knowlesi* infection.

## Conclusion

Phosphoethanolamine-N-methyltransferase has several favourable characteristics supporting its use as a diagnostic target: PMT is absent from the human proteome; low concentration of circulating human antibodies against the *P*. *falciparum* PMT protein were detected; PMT is present throughout the human infecting life-cycle stages; the PMT protein is present at similar or slightly higher concentrations than the current RDT target LDH; the protein is essential for parasite survival and propagation and is considered an antimalarial drug target; and PMT has lower shared identity between *Plasmodium* species orthologues compared to LDH allowing species-specific epitope selection. We describe a common epitope as well as species-specific epitopes for differentiation between *P*. *vivax*, *P*. *falciparum*, *P*. *knowlesi*, *P*. *malariae* and to our knowledge describe the first *P*. *knowlesi* specific protein target with potential for development in malaria diagnostic tests, including RDTs. It is important to assess these antibodies and epitopes with clinical samples and in a RDT format. Though we have evaluated anti-peptide antibodies produced in chickens, it is likely that mouse monoclonal antibodies raised against the same peptides would have the same specificity as that described here. A phage display library expressing single chain variable fragment antibodies is currently being screened to isolate additional antibodies against the PMT protein as has been reported for the *Pf*HRP2 protein [92] to include in our repertoire of potential diagnostic reagents for malaria. PMT appears to be a promising target for evaluation in RDTs and has potential as a *P*. *knowlesi* specific target for consideration as a point-of-care diagnostic test to differentiate and detect *P*. *knowlesi* infection in the Southeast Asia region [8, 21, 22]).
